# Supplementary material for: Digital gene expression analyses of mammary glands from meat ewes naturally infected with clinical mastitis
Source: R Soc Open Sci. 2019 Jul 3;6(7):181604. doi: 10.1098/rsos.181604 (PMC6689637; doi:10.1098/rsos.181604)
Supplement: Table S6 [file rsos181604supp6.docx]

**Journal Name:** Royal Society Open Science

**Digital gene expression analyses of mammary glands from meat ewes naturally infected with clinical mastitis**

**Taotao Li^1^, Jianfeng Gao^1^, Xingxu Zhao^2^ and Youji Ma^1*^**

^1^ College of Animal Science and Technology, and 2 College of Veterinary Medicine, Gansu Agricultural University, Lanzhou, People’s Republic of China

**Table S6.** The differentially expressed genes shared by previous other studies and this study.

| **Transcriptomic technology** | **Tissue samples** | **Common differentially expressed genes with this study** | **References** |
| --- | --- | --- | --- |
| RNA-Seq | Sheep mammary tissues infected with *Mycoplasma agalactiae* | *CXCL13*, *LYZ*, *CD79B*, *CD19*, *S100A8* | [21] |
| RNA-Seq | Sheep milk somatic cells | *PLXNC1*, *CCNYL1*, *PPP4R2* | [38] |
| RNA-Seq | Goat mammary epithelial cells infected with *Mycoplasma agalactiae* | *SERPINE1*, *IL1RN*, *PTX3*, *S100A8*, *S100A9*, *TLR2* | [39] |
| RNA-seq | Cow mammary tissues infected with *Staphylococcus aureus* | *ADAMTS12*, *COL1A1*, *DHRS9*, *GPR68*, *LRRC8C*, *NREP*, *PSPH*, *SOCS3*, *PER2*, *PPP1R1B*, *STAC2*, *UBXN11*, *AOX1*, *SLC24A3*, *PGF*, *GATSL3*, *TUSC5*, *CFAP100*, *MX1*, *FHOD1*, *ADIRF* | [54] |
| Microarray | Cow mammary tissues infected with *Staphylococcus aureus* | *PTX3*, *CSF3R*, *TLR2*, *BCL2A1*, *BIRC3*, *BTG2* | [47] |
| Microarray | Cow mammary tissues infected with coagulase-positive *Staphylococci* | *S100A8*, *S100A9*, *POU2AF1*, *DHRS9*, *SDS*, *IL1RN*, *BTK*, *CXCR2*, *SLC17A9,* *CLEC6A*, *SELE*, *PRKCB*, *LYZ*, *PDPN*, *COL1A1*, *BLNK*, *SRGN*, *CYBB*, *SMPDL3A*, *MSR1*, *SERPINE1*, *BIRC3*, *SLC2A5*, *THBS2*, *RBP1*, *BCL2A1*, *WIPF1*, *BACE2*, *SFRP1*, *NCF2*, *PLAU* | [40] |
| Microarray | Cow mammary tissues infected with coagulase-negative *Staphylococci* | *BCAT1*, *COL1A1*, *GJA1*, *SRGN*, *PRKCB*, *SFRP1*, *BCL2A1*, *EAF2*, *LYZ*, *BTK*, *CXCL13*, *POU2AF1*, *PLAU*, *BACE2*, *SDS*, *SERPINE1*, *WIPF1*, *IL1RN*, *AGTR1* | [40] |
| Microarray | Bovine mammary tissues infected with *Streptococcus uberis* | *S100A9*, *SLAMF7*, *C3* | [41] |
